# FtsZ-Dependent Elongation of a Coccoid Bacterium

**DOI:** 10.1128/mBio.00908-16

**Published:** 2016-09-06

**Authors:** Ana R. Pereira, Jen Hsin, Ewa Król, Andreia C. Tavares, Pierre Flores, Egbert Hoiczyk, Natalie Ng, Alex Dajkovic, Yves V. Brun, Michael S. VanNieuwenhze, Terry Roemer, Rut Carballido-Lopez, Dirk-Jan Scheffers, Kerwyn Casey Huang, Mariana G. Pinho

**Affiliations:** a Bacterial Cell Biology, Instituto de Tecnologia Química e Biológica António Xavier, Universidade Nova de Lisboa, Oeiras, Portugal; b Department of Bioengineering, Stanford University, Stanford, California, USA; c Department of Molecular Microbiology, Groningen Biomolecular Sciences and Biotechnology Institute, University of Groningen, Groningen, The Netherlands; d MICALIS, INRA, AgroParisTech, Université Paris-Saclay, Jouy-en-Josas, France; e W. Harry Feinstone Department of Molecular Microbiology and Immunology, Johns Hopkins Bloomberg School of Public Health, Baltimore, Maryland, USA; f Department of Molecular Biology and Biotechnology, The Krebs Institute, University of Sheffield, Sheffield, United Kingdom; g Department of Biology, Indiana University, Bloomington, Indiana, USA; h Department of Chemistry, Indiana University, Bloomington, Indiana, USA; i Infectious Disease Research, Merck Research Laboratories, Kenilworth, New Jersey, USA; j Department of Microbiology and Immunology, Stanford University School of Medicine, Stanford, California, USA

## Abstract

A mechanistic understanding of the determination and maintenance of the simplest bacterial cell shape, a sphere, remains elusive compared with that of more complex shapes. Cocci seem to lack a dedicated elongation machinery, and a spherical shape has been considered an evolutionary dead-end morphology, as a transition from a spherical to a rod-like shape has never been observed in bacteria. Here we show that a *Staphylococcus aureus* mutant (M5) expressing the *ftsZ*^G193D^ allele exhibits elongated cells. Molecular dynamics simulations and *in vitro* studies indicate that FtsZ^G193D^ filaments are more twisted and shorter than wild-type filaments. *In vivo*, M5 cell wall deposition is initiated asymmetrically, only on one side of the cell, and progresses into a helical pattern rather than into a constricting ring as in wild-type cells. This helical pattern of wall insertion leads to elongation, as in rod-shaped cells. Thus, structural flexibility of FtsZ filaments can result in an FtsZ-dependent mechanism for generating elongated cells from cocci.

## IMPORTANCE

The mechanisms by which bacteria generate and maintain even the simplest cell shape remain an elusive but fundamental question in microbiology. In the absence of examples of coccus-to-rod transitions, the spherical shape has been suggested to be an evolutionary dead end in morphogenesis. We describe the first observation of the generation of elongated cells from truly spherical cocci, occurring in a *Staphylococcus aureus* mutant containing a single point mutation in its genome, in the gene encoding the bacterial tubulin homologue FtsZ. We demonstrate that FtsZ-dependent cell elongation is possible, even in the absence of dedicated elongation machinery.

## INTRODUCTION

Cell morphology is a distinctive characteristic of bacterial species and has been used extensively for their classification (1). In most bacteria, cell shape is maintained by the peptidoglycan (PG), a macromolecular polymer that surrounds the cell, confers mechanical strength and resists expansion due to turgor pressure. Spatial and temporal control of PG synthesis and remodeling is critical for defining and maintaining a particular shape (2, 3). Nevertheless, the mechanisms by which cell shape diversity is generated remain largely elusive.

Although a myriad of shapes within the bacterial kingdom has been described, most of the well-studied species are rods, ovococci, or cocci. These shapes result from different mechanisms of cell wall growth and from the presence of various cytoskeletal elements. The best-studied rod-shaped bacteria maintain their characteristic shape through two PG synthesis modes coordinated by major cytoskeletal elements: elongation of the sidewall, coordinated mainly by the actin homologue MreB, and placement of a crosswall (septum) during division, coordinated by the tubulin homologue FtsZ (4). FtsZ is a self-activating GTPase that forms a ring (the Z ring) at the future site of division, which recruits several other cell division and PG synthesis proteins that drive septum formation (5). While the division mechanism is conserved in most bacteria, elongation modes are variable. In rod-shaped species that express MreB homologues, such as *Bacillus subtilis* and *Escherichia coli*, MreB-associated PG-synthesizing complexes assemble in dynamic patches underneath the cell membrane and promote the coordinated insertion of PG throughout the cell periphery to result in cell elongation (6–8). Ovococcoid bacteria do not possess MreB homologues, but they also elongate, albeit slightly, by the so-called peripheral PG synthesis that occurs in close proximity to the division site, coordinated by the Z ring (3, 9). Spherical cocci lack dedicated elongation machinery and divide using an FtsZ-dependent system, placing new cell wall mostly at the mid-cell division septum (10).

Interestingly, phylogenetic analyses suggest that modern cocci evolved from rod-shaped bacteria, possibly through the loss of dedicated elongation machinery (11). An example of this rod-to-coccus transition by loss of specific genes was recently reported for *Neisseria meningitidis* and *Moraxella catarrhalis* during adaption to the nasopharynx niche (12). Accordingly, rod-shaped bacteria can acquire a spherical shape upon the inactivation of elongation-specific cytoskeletal proteins or PG synthesis enzymes (13–18). As for the opposite coccus-to-rod transition, ovococci can generate more elongated cells upon inhibition of septation, despite the absence of MreB (19, 20). However, to the best of our knowledge, there are no reports of elongation in otherwise spherical bacteria. Spherical morphology is therefore viewed as an evolutionary dead end from the perspective of cell shape (11).

In this report, we describe the first mechanism to convert spherical *Staphylococcus aureus* cells into elongated cells. This behavior was observed in a mutant previously isolated during the screening of methicillin-resistant *S. aureus* strain COL for resistance to PC190723, an antibiotic that inhibits cell division by targeting FtsZ (21, 22). Genome sequencing of the mutant revealed a single point mutation (G193D) in FtsZ (22). On the basis of our findings obtained with a combination of superresolution microscopy, electron microscopy, molecular dynamics (MD), and biochemical analyses of the FtsZ mutant protein, we propose an FtsZ-dependent mechanism for the morphogenesis of elongated *S. aureus* cells.

## RESULTS

### FtsZ^G193D^ mutation leads to elongated cells in *S. aureus.*

**FIG 1:**
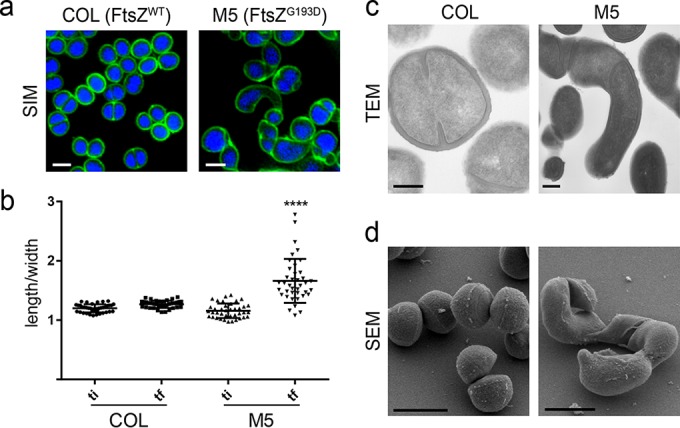
The FtsZ^G193D^ mutation leads to *S. aureus* cell elongation. (a) SIM images of wild-type COL (left) and FtsZ^G193D^ mutant M5 (right) cells labeled with the cell wall dye Van-FL (green) and the DNA dye Hoechst 33342 (blue). Scale bar: 1 µm. (b) The ratio of the longer to the shorter axis was calculated at two time points during the cell cycle, an initial time point (ti) when cells had a round shape and a final time point (tf) when cells were most elongated, prior to splitting of the mother cell. M5 cells elongate significantly more during the cell cycle than COL wild-type cells (*P* < 0.001). (c) Transmission electron micrographs (TEM) of thin sections of COL and M5 cells. Scale bars: 200 nm. (d) Scanning electron microscopy (SEM) images of COL and M5 cells. Scale bars: 1 µm.

*S. aureus* cells are approximately spherical, and there are no previous reports of a sphere-to-rod transition in cocci. Putative mechanisms to generate elongated cells of *S. aureus* include expressing an actin-like cytoskeleton or inhibiting cell division or septal cell wall synthesis. However, expression of *B. subtilis* MreB (23) or Mbl (our unpublished observations) does not result in elongated *S. aureus* cells. Similarly, mutations that reduce FtsZ function can produce enlarged spherical cells (24, 25), showing that the peripheral PG synthesis that occurs in *S. aureus* does not support elongation (10). Serendipitously, while characterizing PC190723-resistant *S. aureus* mutant M5 (22), which carries a G-to-D substitution at the 193rd residue of FtsZ within helix 7, we noticed the presence of cells that were not spherical. In order to examine the shape alterations of this mutant in more detail, we labeled the COL wild-type and M5 mutant strains with fluorescently modified vancomycin (Van-FL, which labels the entire cell wall in *S. aureus*) and the DNA dye Hoechst 33342. Superresolution imaging of the labeled cells by structured illumination microscopy (SIM) confirmed the presence of cells with altered morphology, including curved elongated cells (Fig. 1a). Despite the substantial morphological changes, M5 mutant cells were able to grow reasonably well, albeit more slowly than parental strain COL grown in batch culture (47 versus 25 min, respectively, see Fig. S1 in the supplemental material).

To quantitatively evaluate elongation, COL and M5 cells were stained with the membrane dye Nile red, and cell shape alterations were monitored over the cell cycle by time-lapse microscopy (see Fig. S2a and b in the supplemental material). Measurements of the longer (cell length) and shorter (cell width) axes of both M5 and COL cells (*n* > 50 cells) showed that the length-to-width ratio is significantly increased in M5 mutant cells (Fig. 1b). Membrane labeling also confirmed that cells underwent true elongation, not division without cell separation, as long cells were devoid of septa (see Fig. S2c). Elongation of *S. aureus* M5 cells was further confirmed by transmission electron microscopy and scanning electron microscopy (Fig. 1c and d). Changes in morphology were not due to altered FtsZ expression levels, as the total amounts of FtsZ^G193D^ and FtsZ^WT^ in M5 and COL cells, respectively, were similar, as determined by Western blotting (see Fig. S3 in the supplemental material).

Since G193 is well conserved across FtsZ proteins, we wondered if introduction of the FtsZ^G193D^ mutation into a rod-shaped bacterium would also have an effect on cell shape, generating longer or curved cells. We attempted to replace the native *ftsZ* allele in *B. subtilis* with the allele encoding the G193D mutation. However, in agreement with a recent report (25), we were unable to obtain viable *B. subtilis* colonies expressing *ftsZ*^G193D^ as the sole copy of *ftsZ* on the chromosome, suggesting that the mutation is lethal in *B. subtilis*. We then constructed *B. subtilis* strain PF20 with both *ftsZ*^WT^ and *ftsZ*^G193D^ alleles under the control of two different inducible promoters (see Fig. S4a in the supplemental material). This strain failed to form colonies when *ftsZ*^G193D^ was the only allele expressed, confirming that the mutation is lethal in *B. subtilis* (see Fig. S4b and c). Consistently, FtsZ^G193D^ was delocalized and did not form Z rings, indicating that it is unable to promote cell division (see Fig. S4d).

### The G193D mutation leads to shorter, twisted FtsZ polymers.

**FIG 2:**
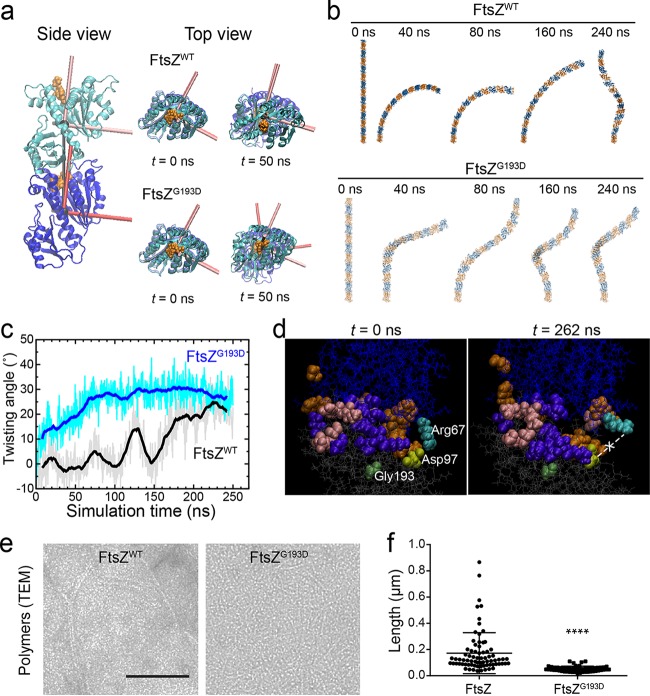
FtsZ^G193D^ produces twisted and shorter polymers. (a) MD simulations of dimers of *S. aureus* FtsZ^WT^ (PDB code 3VO8) and FtsZ^G193D^ reveal a propensity for twist in FtsZ^G193D^ dimers. Simulations were initialized as straight dimers at *t* = 0 (side view). The top view highlights the development of twist between the principal axes of the two FtsZ^G193D^ subunits at *t* = 50 ns. (b) Shown are filaments of 14 subunits constructed by extrapolating the dimer interface from time points during the MD simulations. The FtsZ^WT^ dimer bent but remained untwisted for the first ~100 ns. (c) The FtsZ^G193D^ dimer rapidly adopted a substantial degree of twist, while the FtsZ^WT^ dimer acquired twist much later in the simulation. (d) Snapshots at the beginning and end of the FtsZ^WT^ dimer simulation, in which residues are colored if they interact (defined as being within 5 Å of another residue) with the opposite subunit at any point during the simulation. Colors: orange, specific to the nontwisted state of the wild type, with an interaction in the first 100 ns and no interaction after 150 ns (and no interaction throughout the FtsZ^G193D^ simulation); purple, generally present in twisted states (always interacting in FtsZ^G193D^ and after 150 ns for FtsZ^WT^); pink, specific to FtsZ^G193D^. The salt bridge between Asp97 (light green) and Arg67 (cyan) became broken at *t* = 262 ns. Gly193 is dark green. (e) Polymers of *S. aureus* FtsZ^WT^ (left) and FtsZ^G193D^ (right) imaged by transmission electron microscopy (TEM). Scale bar: 100 nm. (f) Measurements of FtsZ^WT^ and FtsZ^G193D^ polymer (*n* >70) lengths in the TEM images show that FtsZ^G193D^ polymers are significantly shorter than FtsZ^WT^ polymers (*P* < 0.0001).

The FtsZ filament binding interface has been shown to have the capacity to rearrange itself to accommodate different degrees of bending (26, 27). We wondered whether the G193D mutation might cause major structural rearrangements in FtsZ, resulting in misassembled and/or mislocalized FtsZ rings that could produce the morphological phenotypes of M5 cells. We carried out all-atom MD simulations of *S. aureus* FtsZ^WT^ and FtsZ^G193D^ dimers to assay whether the structure of the subunits and/or the polymers would likely be affected by the mutation (Fig. 2a). Simulations were initiated from a straight, untwisted state, as shown by repeating the dimer interface to create a long polymer (Fig. 2a and b). However, FtsZ^G193D^ homodimers reproducibly adopted a twist of >15° between the two monomers within the first 50 ns of simulation, while the FtsZ^WT^ dimer remained untwisted for >100 ns (Fig. 2a to c). This predicted twist could have two effects *in vivo*: (i) the spatial pattern of membrane binding could be more likely to follow a helix rather than the normal Z ring, and (ii) such twisting could impair the formation or stability of FtsZ polymers.

To examine how the binding interface between the subunits reorganized upon twisting, we identified all of the pairs of amino acids that spanned both subunits and whose closest atoms lay within 5 Å. The number of such pairs ranged between 13 and 24 and between 14 and 26 during the simulation of the FtsZ^WT^ and FtsZ^G193D^ dimers, respectively. Such interactions could be classified into three groups: those that are present early in the FtsZ^WT^ dimer simulation and never in FtsZ^G193D^ (representing a nontwisted state), those that are always present in FtsZ^G193D^ and only later in FtsZ^WT^ (general to twisted states), and those that are present only in FtsZ^G193D^ (specific to the twisted state of the mutant) (see Fig. S5a in the supplemental material). The residues in each of these groups were spatially clustered (Fig. 2d), indicating that it is not merely fluctuations of the interface that generate the interactions and that there are binding interfaces specific to FtsZ^WT^ and FtsZ^G193D^, with FtsZ^G193D^ having interactions that do not exist in the FtsZ^WT^ twisted state.

There was also a single interaction, between the arginine at position 67 on the top FtsZ subunit and the aspartic acid at position 97 on the bottom subunit, that followed dynamics similar to those of the twist angle in the FtsZ^WT^ dimer (Fig. 2d). This salt bridge was connected for the first 100 ns, broke, reformed briefly, and then was broken for the remainder of the simulation (see Fig. S5b). Upon closer examination, the arginine appeared to form a swinging lever that was initially bound to the aspartic acid but swung upward during the periods of twisting. Glycine 193 is part of a helix on the bottom subunit that connects to residues 202 to 204 and Asp97, suggesting that the mutation to aspartic acid (G193D) allosterically disrupts the salt bridge and potentially destabilizes the interface.

We next purified *S. aureus* FtsZ^WT^ and FtsZ^G193D^ and studied *in vitro* polymer formation by transmission electron microscopy. Although FtsZ^G193D^ was capable of polymerization, the resulting polymers were significantly shorter than those made by FtsZ^WT^ under the same conditions (Fig. 2e and f), suggesting that FtsZ^G193D^ polymers could potentially be less stable, as predicted by the MD simulations. Changes in FtsZ polymer stability, especially those mediated by regulatory proteins, are often linked to changes in the GTP hydrolysis activity of FtsZ (28). However, there was no significant difference between the GTP hydrolysis rates of FtsZ^WT^ and FtsZ^G193D^ (0.14 ± 0.02 and 0.15 ± 0.03 mol of phosphate released per mol of FtsZ per min, respectively; see Fig. S6 in the supplemental material).

### Z rings are misplaced in *S. aureus* FtsZ^G193D^ mutant cells.

**FIG 3:**
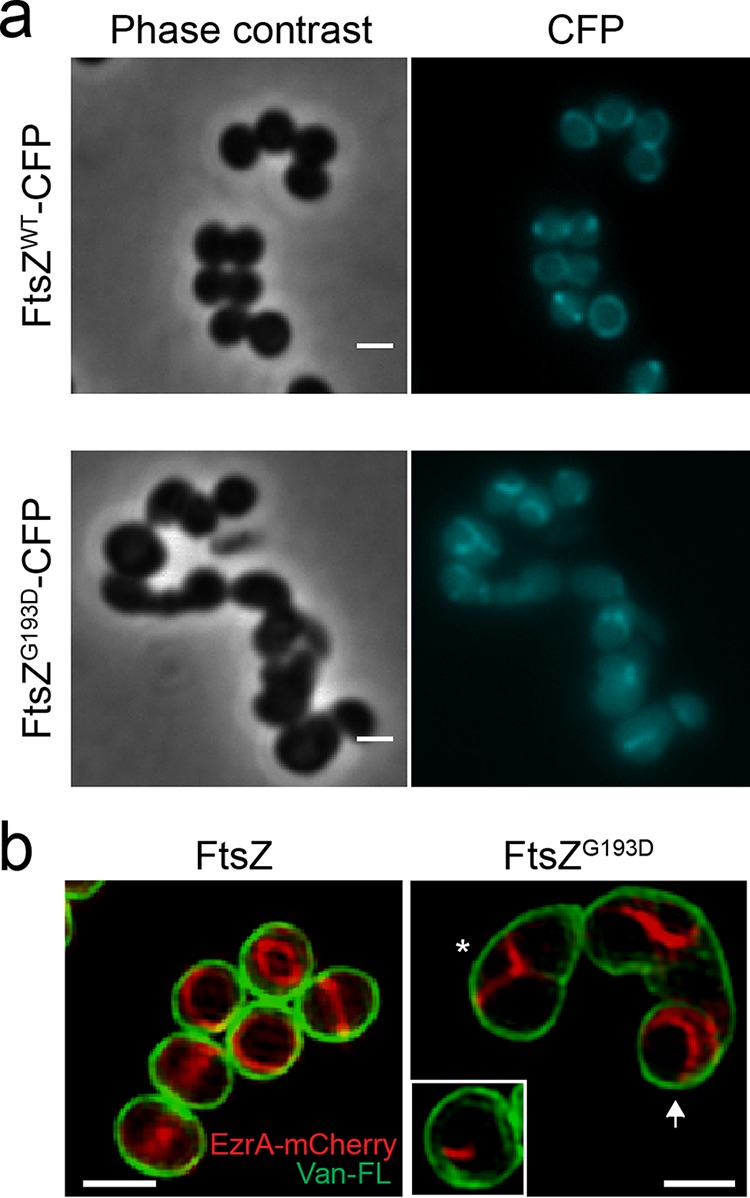
Fluorescent fusions with FtsZ and EzrA lose mid-cell localization in M5 cells. (a) Localization of FtsZ^WT^-CFP in BCBAJ020 cells (top) and FtsZ^G193D^-CFP in BCBRP005 cells (bottom). Phase-contrast (left) and epifluorescence (right) images are shown. (b) SIM images of EzrA-mCherry (red) and cell wall labeled with Van-FL (green) in BCBAJ012 cells expressing FtsZ^WT^ (left) and in BCBRP006 cells expressing FtsZ^G193D^ (right). In the presence of FtsZ^G193D^, EzrA-mCherry fails to localize as a mid-cell ring. Instead, structures assemble on only one side of the cell (white box) or in Y patterns compatible with one-turn helical structures (asterisk). More elongated BCBRP006 cells show EzrA-mCherry localized toward the poles of the cell (white arrow). Scale bars: 1 µm.

Our simulations predict a propensity for twisting in FtsZ^G193D^ homodimers (Fig. 2a and b) that could result in polymers that assemble into extended helical structures instead of a ring. Interestingly, helical FtsZ structures have been observed both in *E. coli* and in *B. subtilis* (29–32). Therefore, we determined the localization of a fluorescent protein fusion of FtsZ^G193D^ in live *S. aureus* cells. Given that available fusions with *S. aureus* FtsZ are not fully functional (33), we expressed FtsZ^G193D^ tagged with cyan fluorescent protein (CFP) at the C terminus from the ectopic spa locus, under control of the P_spac_ promoter (33). We then replaced the wild-type *ftsZ* gene at the native chromosomal locus with an allele containing the *ftsZ* G578A mutation that encodes the FtsZ^G193D^ protein, thereby generating strain BCBRP005, which recapitulates the morphological phenotypes of M5. While FtsZ^WT^-CFP localized as a ring at mid-cell (Fig. 3a, top) as previously described (33), FtsZ^G193D^-CFP localized in structures that extend along the cell in different orientations (Fig. 3a, bottom). These localization patterns were confirmed by immunofluorescence microscopy in the original COL and M5 strains (see Fig. S7a in the supplemental material).

As fluorescent protein fusions with FtsZ are not fully functional and the weak FtsZ-CFP fluorescence is incompatible with SIM, we used EzrA-mCherry as a proxy for Z-ring localization. EzrA, an early component of the divisome, is a regulator of FtsZ polymerization and directly interacts with FtsZ (34–38). We replaced the native *ftsZ* gene with the *ftsZ*^G193D^ allele in strain BCBAJ012, which expresses EzrA-mCherry as the sole copy of EzrA (37), to create strain BCBRP006. These cells had a morphological phenotype similar to that of M5 cells (Fig. 3b), confirming that FtsZ^G193D^ is sufficient to cause cell elongation. In the presence of the FtsZ^G193D^ mutation, EzrA-mCherry no longer localized only as a mid-cell ring but instead frequently assembled at only one side of cells that were beginning to elongate (white box inset in Fig. 3b) or in a Y shape in cells that were partially elongated (asterisk in Fig. 3b). As FtsZ^G193D^ mutant cells elongated further, EzrA structures became localized toward the “poles” of the cells (white arrow in Fig. 3b). One-turn helical structures would appear as such Y shapes in two-dimensional microscopy images. Because of photobleaching, we were unable to visualize the EzrA-mCherry Y shapes in three dimensions by SIM. We therefore acquired Z-stacks of SIM images of M5 cells labeled with Van-FL, which labels the cell wall, including the nascent septum, confirming that Y shapes along the membrane corresponded to one-turn helical structures (see Video S1 in the supplemental material).

### FtsZ^G193D^ structures recruit PG synthesis enzymes.

Wild-type FtsZ rings recruit PG synthesis proteins, namely, penicillin-binding proteins (PBPs), that catalyze the synthesis of glycan strands (transglycosylation) and their cross-linking via peptide bridges (transpeptidation). These activities are required for the formation of the division septum, which is perpendicular to the cell surface and extends inward, across the mother cell. If misplaced FtsZ^G193D^ structures also recruit PBPs, then spatially misplaced incorporation of septal PG could follow, which could explain cell elongation.

*S. aureus* has four native PBPs, of which PBP2 is the only bifunctional enzyme, capable of catalyzing both transpeptidation and transglycosylation reactions (39–41). PBP2 localizes to the septum in an FtsZ-dependent manner, and in the absence of FtsZ, PBP2 becomes dispersed over the entire cell surface (24). To localize PBP2 in the presence of FtsZ^G193D^, wild-type *ftsZ* was replaced with the *ftsZ*^G193D^ allele in the background of strain BCBPM073 (22), which expresses superfast green fluorescent protein (sGFP)-PBP2 as the sole copy of PBP2. In FtsZ^G193D^ mutant cells, sGFP-PBP2 no longer formed septal rings at mid-cell, but rather than becoming dispersed, it had a localization pattern similar to that of FtsZ^G193D^ structures (see Fig. S7B). On the basis of this, we infer that FtsZ^G193D^ filaments are still capable of recruiting PBP2 and possibly other PG synthesis proteins, indicating that FtsZ^G193D^ filaments are competent to promote cell wall synthesis.

**FIG 4:**
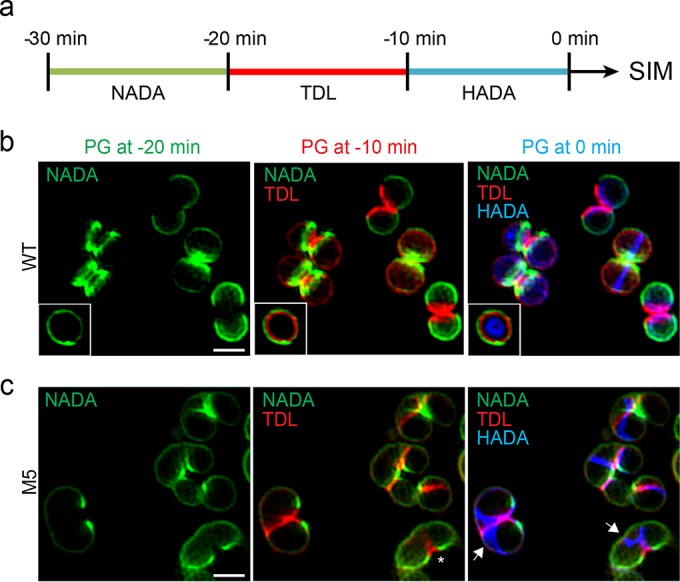
PG incorporation dynamics suggest a new mode of cell growth in *S. aureus* FtsZ^G193D^ cells. (a) Schematic representation of pulse-labeling with FDAAs. Cells were labeled with three sequential 10-min pulses of NADA (green), TDL (red), and HADA (blue) and observed by SIM. The same cell imaged in three different channels therefore shows the PG synthesized 30 to 20 min (green), 20 to 10 min (red), and 10 to 0 min (blue) prior to imaging. (b) In COL cells, septum synthesis initiates as a symmetric ring at mid-cell that constricts over time, concomitant with concentric PG synthesis (white box). (c) In M5 cells, PG synthesis occurs in two steps. First, PG is asymmetrically incorporated on only one side of a cell, eventually leading to local splitting (asterisks). Second, PG is inserted along a Y-shaped pattern compatible with a one-turn helical path (blue fluorescence, white arrows). Scale bars: 1 µm.

We therefore examined PG incorporation over the cell cycle by sequentially labeling COL and M5 cells with fluorescent d-amino acids (FDAAs) derivatized with fluorophores of different colors, which are incorporated at sites of active PG synthesis (42), thereby creating a virtual time-lapse image of PG synthesis locations. COL and M5 cells were labeled with green 7-nitrobenzo-2-oxa-1,3-diazole–amino-d-alanine (NADA) for a short 10-min pulse, followed by a 10-min pulse with the red d-amino acid 6-carboxytetramethylrhodamine–d-lysine (TDL), and finally by a 10-min pulse with the blue d-amino acid 7-hydroxycoumarin–amino-d-alanine (HADA). We then imaged the subcellular localization of PG synthesis by SIM (Fig. 4). As expected, septal PG synthesis progression in wild-type COL cells occurred in continuous mid-cell rings that constricted over time and finally split, giving rise to new daughter cells (Fig. 4b). Note that *S. aureus* divides into three perpendicular planes over successive division cycles and therefore sequential division planes are orthogonal (3, 33). In contrast, in M5 cells, PG was first incorporated only on one side of the cell (asterisk in Fig. 4c) and then progressed along the periphery of the cell, in a pattern compatible with a helical structure (blue fluorescence, white arrows in Fig. 4c).

### FtsZ^G193D^ leads to a new mode of cell division for *S. aureus.*

**FIG 5:**
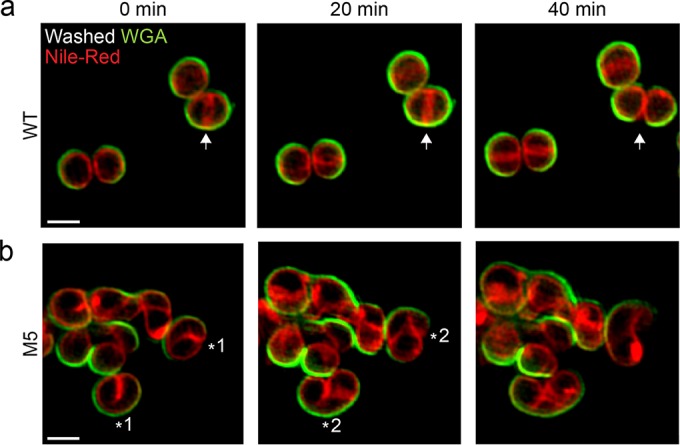
SIM time-lapse imaging reveals the mode of elongation of M5 cells. *S. aureus* COL (FtsZ^WT^) and M5 (FtsZ^G193D^) cells were stained with the peripheral cell wall dye WGA-488 (green). Unbound dye was washed away, labeled cells were stained with the membrane dye Nile red, and growth was monitored by SIM. (a) COL wild-type (WT) cells synthesized a ring-like septum at mid-cell (arrow at 0 min) that constricts and eventually splits, giving rise to two daughter cells (arrow at 40 min). (b) M5 mutant cells initially synthesized an asymmetric septum on only one side of the cell (asterisk 1), which later developed into a pattern compatible with a one-turn helical structure (asterisk 2) and led to elongation. Scale bars: 1 µm.

To observe the fate of cells with different modes of PG insertion, COL or M5 cells were labeled with the wheat germ agglutinin-Alexa Fluor 488 conjugate dye (WGA-Alexa488), which labels nonseptal cell wall. Labeled cells were then placed on an agarose pad containing growth medium and the membrane dye Nile red and imaged in time-lapse mode by SIM (Fig. 5). As previously observed, wild-type COL cells displayed a mid-cell septum that constricted and split to generate approximately one-third of the surface of each new daughter cell (10) (Fig. 5a, white arrow). In M5 mutant cells, septum synthesis did not initiate as a homogeneous ring encircling the cell. Instead, during early stages of division, septum formation progressed from only one side of the cell (Fig. 5b, asterisk 1). The region where the asymmetric septum first formed proceeded into local splitting of the cell, increasing the cell surface in a manner similar to that of wild-type *S. aureus* cells upon splitting of the division septum (compare the bottom cell labeled with an asterisk in the left and middle parts of Fig. 5b). At a later stage, the asymmetric septum extended, forming a structure compatible with a one-turn helix (Fig. 5b, asterisk 2). Regions where PG was inserted along this pattern resulted in cell elongation without splitting, via increase of the surface area of the peripheral wall (see also blue dye indicated by white arrows of Fig. 4c), reminiscent of elongation in rod-shaped bacteria.

## DISCUSSION

**FIG 6:**
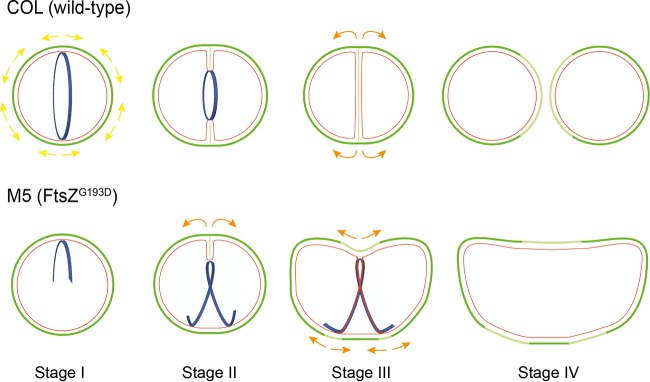
Model of *S. aureus* M5 cell elongation. Schematic of cell division events in wild-type COL (top) and M5 (bottom) cells. The images show old cell wall (dark green), new cell wall (light green), membrane (red), and FtsZ structures (blue). Yellow/orange arrows correspond to surface expansion. In wild-type cells (top), FtsZ assembles as a ring at mid-cell and recruits PG synthesis enzymes, leading to the incorporation of new PG at mid-cell. New PG is also inserted along the cell periphery, leading to cell surface expansion (yellow arrows). Splitting of the septum generates ~1/3 of the cell surface of each of the new daughter cells, thereby increasing the total surface area exposed to the external milieu. M5 cells (bottom) expressing FtsZ^G193D^ rather than FtsZ^WT^ incorporate PG in two steps. First, PG deposition occurs on only one side of the cell (asymmetric septum, division stage I), which will initiate local splitting following septal constriction (stage II) that leads to surface area expansion only on that side of the cell (orange arrows). Second, PG deposition is directed by FtsZ along a partial helical path, leading to further surface expansion resembling elongation of rod-shaped cells (stage III). Thus, M5 cells combine a coccus-like (septal) mode of PG incorporation (stage II) with a rod-like elongation mode (stages III and IV), both of which are coordinated by FtsZ.

In this work, we provide the first report of elongation in spherical bacteria, i.e., true cocci, which have no dedicated PG synthesis elongation machinery. Wild-type *S. aureus* cells synthesize cell wall mostly at the septum, which, upon splitting, generates approximately one-third of the cell surface of the new daughter cells and thereby constitutes the largest cell surface increase event in the staphylococcal cell cycle (10). The FtsZ^G193D^ mutation in M5 cells results in shorter and twisted FtsZ polymers that fail to localize as a ring at mid-cell. As a consequence, the typical mid-cell localization of PG synthesis is lost and during cell division, septum synthesis is initiated in a helical pattern. We propose that helical FtsZ^G193D^ structures direct PG synthesis, initially localized asymmetrically to only one side of the cell (Fig. 6, division stage I). This mode of synthesis is similar to septal synthesis in wild-type *S. aureus* cells (Fig. 6, top, division stage I and II) and is followed by local splitting, generating an increase in surface area as the septal wall is converted into peripheral wall and exposed to the external milieu (Fig. 6, bottom, from stage II to stage III, orange arrows). PG incorporation then occurs in a partial helical pattern (Fig. 6, division stage III) around the cell periphery, leading to cell elongation (Fig. 6, division stage IV). This elongation is not equivalent to the dispersed PG synthesis all over the cell surface that we recently described for wild-type cells (10), which is not dependent on a cytoskeletal element. Instead, it is reminiscent of MreB-dependent sidewall elongation in rod-shaped bacteria, since this elongation PG synthesis is localized and directed by a cytoskeletal element, namely, FtsZ filaments.

Interestingly, the M5 mutant was previously shown to have attenuated virulence in a murine model (22). Our observation that M5 mutant cells exhibit an elongated morphology is therefore in agreement with recently suggested hypotheses that a spherical shape or smaller size may constitute a selective advantage for pathogens that colonize the nasopharynx, the ecological niche of *S. aureus*, possibly by a decrease in the cell surface area exposed to immune system (12, 43).

## MATERIALS AND METHODS

### Bacterial strains and growth conditions.

All of the strains and plasmids used in this study are listed in Table S1A in the supplemental material. The sequences of the primers used are listed in Table S1B. See Text S1 in the supplemental material for more information regarding the growth conditions, strain construction, and techniques used for GTP hydrolysis assay, immunofluorescence assay, Western blotting, protein purification, and microscopy in this study.

### Measurements of cellular aspect ratios.

*S. aureus* overnight cultures were diluted 1:200 in fresh medium and incubated at 37°C. One milliliter of each culture in exponential phase (optical density at 600 nm [OD_600_] of 0.6) was incubated with the membrane dye Nile red (10 µg ml^−1^), at 37°C for 5 min with shaking. Cells were harvested and resuspended, and 1 µl was placed on a pad of 1.2% agarose in tryptic soy broth (TSB) mounted on a microscope slide. Epifluorescence images were recorded every 5 min with a Zeiss Axio Observer.Z1 microscope equipped with a Photometrics CoolSNAP HQ2 camera (Roper Scientific) using ZEN blue software. Fifty round cells of each strain were chosen, and the longer (length) and shorter (width) axes were measured at the beginning of the experiment and at the stage of maximum elongation (end of the cell cycle, just prior to cell splitting). The length-to-width ratios at both time points were then calculated. Statistical analyses were performed in GraphPad Prism 5 by using the Mann-Whitney test for nonnormal distributions.

### MD simulations.

All simulations were performed with the MD package NAMD (44) with the CHARMM27 force field (45, 46), including CMAP corrections (47). Water molecules were described with the TIP3P model (48). Long-range electrostatic forces were evaluated by means of the particle mesh Ewald summation approach with a grid spacing of <1 Å. An integration time step of 2 fs was used (49). Bonded terms and short-range, nonbonded terms were evaluated at every time step, and long-range electrostatics were evaluated at every other time step. A constant temperature (*T* = 310 K) was maintained by using Langevin dynamics (50), with a damping coefficient of 1.0 ps^−1^. A constant pressure of 1 atm was enforced by using the Langevin piston algorithm (51) with a decay period of 200 fs and a time constant of 50 fs. Simulations were initialized from the *S. aureus* GDP-bound FtsZ dimer structure (Protein Data Bank [PDB] code 3VO8) (52). Water and neutralizing ions were added around the FtsZ dimer. Force field parameters for GTP and GDP molecules were taken from reference 27. All simulations with FtsZ dimers contained approximately 211,000 atoms. Setup, analysis, and rendering of the simulation systems were performed with the software VMD (53).

### EzrA-mCherry localization by SIM.

Exponentially growing cultures of strains BCBAJ012 and BCBRP006 (OD_600_ of 0.6) were labeled with a 1:1 (vol/vol) mixture of the cell wall dye Van-FL (1 μg ml^−1^; Invitrogen) and nonfluorescent vancomycin (1 µg ml^−1^; Sigma) for 5 min at room temperature with shaking. Cultures were pelleted, resuspended in 20 µl of phosphate-buffered saline (PBS), and placed on top of a thin layer of 1.2% agarose in PBS mounted on a microscope slide. Imaging was performed by SIM with the 561-nm laser at maximal power for mCherry observation and the 488-nm laser at 20% maximal power for Van-FL.

### Cell wall staining with FDAAs.

Pulse-labeling experiments were performed essentially as previously described (42). In brief, the COL and M5 strains were incubated overnight at 37°C in TSB, diluted 1:200 in fresh medium, and incubated at 37°C. At an OD_600_ of 0.8, 500 µl of each culture was harvested by centrifugation, resuspended in 150 µl of supernatant, and labeled with 25 µM green NADA (42, 54). Labeling was performed for 10 min at 37°C with shaking. Unbound NADA was removed by washing with 200 µl of PBS, and NADA-labeled cultures were resuspended in 150 µl of spent medium. A second labeling step was then performed with 25 µM red TDL (42, 54) for 10 min at 37°C with shaking. Unbound TDL was removed by washing with 200 µl of PBS, and NADA-TDL-labeled cultures were resuspended in 150 µl of spent medium. A third labeling step was performed with 25 µM blue HADA for 10 min at 37°C with shaking. Cultures were then washed with 200 µl of PBS and resuspended in 50 µl of PBS, and 1 µl was placed on a microscopy slide covered with a thin layer of 1.2% agarose in PBS. NADA, TDL, and HADA fluorescence was imaged by SIM with 50% of the maximal laser power at 488, 561, and 405 nm, respectively, with an exposure time of 30 ms.

### SIM time-lapse imaging.

To observe individual cell division events, exponentially growing cultures (OD_600_ of 0.8) of *S. aureus* wild-type COL or M5 were first stained with the peripheral cell wall dye WGA-Alexa488 (Invitrogen) at a final concentration of 2 µg ml^−1^ for 5 min at 37°C with shaking. Unbound dye was removed by washing cells with TSB. Cells were then placed on an agarose pad mounted on a microscope slide containing 50% TSB in PBS and the membrane dye Nile red (10 µg ml^−1^). Cell growth was tracked by SIM from images acquired every 20 min with 20-ms exposures at 10% of the maximal 488-nm laser power and 20% of the maximal 561-nm laser power.

## SUPPLEMENTAL MATERIAL

Figure S1  Analysis of COL wild-type and M5 mutant culture growth at 37°C. (a) Growth of COL and M5 cultures in TSB medium was monitored by recording the OD_600_ every hour. (b) The number of CFUs was also determined throughout the growth curve by plating appropriate dilutions of the growing COL and M5 cultures on solid tryptic soy agar. The graph shows the log_10_ number of CFU/ml versus time. Download Figure S1, TIF file, 0.2 MB

Figure S2  M5 mutant cell elongation observed by time-lapse microscopy and SIM. (a, b) Shown is time-lapse microscopy of COL wild-type (a) and M5 mutant (b) cells with examples of cells used to measure width/length ratios from round to elongated shapes plotted in Fig. 1b. Overlaid phase-contrast and Nile red-stained images are shown. Images were taken every 5 min. (c) Examples of elongated M5 cells without a septa stained with the membrane dye Nile red and observed by SIM. Scale bars: 1 µm. Download Figure S2, TIF file, 1.8 MB

Figure S3  FtsZ^G193D^ levels in M5 cells are similar to FtsZ^WT^ levels in COL cells. Western blot analysis shows similar levels of FtsZ protein in COL and M5 cells. Twenty micrograms (first two lanes) or 10 µg (last two lanes) of total protein in crude cell extracts was loaded into the gel. PBP2 was used as an internal control. Download Figure S3, TIF file, 0.1 MB

Figure S4  The FtsZ^G193D^ mutation renders FtsZ nonfunctional in *B. subtilis*. (a) Schematic representation of the genotypes of strains PF19, PF20, PF21, and PF22 used in this study. (b) Strains PF19 and PF20 were streaked onto plates supplemented with either 0.5% (wt/vol) xylose (left) or 10 µM isopropyl-β-d-thiogalactopyranoside (IPTG) (right) to induce the expression of the *ftsZ* alleles controlled by the respective promoters, as indicated in panel a. No differences in growth between strains PF20 and PF19 expressing only FtsZ^WT^ (in the presence only of xylose) were observed. However, strain PF20 was not viable when expressing FtsZ^G193D^ as the only source of FtsZ in the cell (in the presence only of IPTG). (c) Growth of PF20 and PF19 was measured in either LB plus xylose (0.2% [wt/vol], diamonds) or LB plus IPTG (100 µM, squares), confirming that cells expressing only FtsZ^G193D^ are not viable. (d) FtsZ^G193D^-GFP localizes as a diffuse cytoplasmic signal in *B. subtilis* and cannot form Z rings. Cells of strains PF21 (left) and PF22 (right) were grown in LB plus IPTG (100 µM) to express FtsZ^WT^ or FtsZ^G193D^, respectively, mounted on an agarose pad, and imaged by epifluorescence microscopy. Scale bars: 2 µm. Download Figure S4, TIF file, 1.3 MB

Figure S5  Interfacial interactions differ in FtsZ^WT^ and FtsZ^G193D^ and between the nontwisted and twisted states. (a) All of the residues that interact with the opposite subunit (defined as being within 5 Å of another residue) were identified in each frame of the simulations. Shown are the interactions in 12.5-ns blocks. Black, specific to the nontwisted state of the wild type, with an interaction in the first 100 ns and no interaction after 150 ns (and no interaction throughout the FtsZ^G193D^ simulation); purple, generally present in twisted states (always interacting in FtsZ^G193D^ and after 150 ns for FtsZ^WT^); blue, specific to FtsZ^G193D^. Red oval highlights Asp97. (b) Shown is the distance between the centers of mass of Arg67 and Asp97 spanning the dimer interface. The salt bridge was rapidly broken in the FtsZ^G193D^ dimer simulation. In the FtsZ^WT^ dimer simulation, the salt bridge briefly destabilized at *t* = ~100 ns and then broke for the remainder of the simulation at around *t* = 120 ns, mimicking the trajectory of polymer twist (Fig. 2b). Download Figure S5, TIF file, 0.3 MB

Figure S6  The FtsZ^G193D^ mutation does not affect GTP hydrolysis. Shown is the average number of phosphate molecules released per FtsZ^WT^ (circles) or FtsZ^G193D^ (squares) molecule. Average values are from four independent assays, and error bars represent standard deviations. Download Figure S6, TIF file, 0.1 MB

Figure S7  FtsZ^G193D^ and PBP2 do not form a mid-cell ring in *S. aureus*. (a) Phase-contrast and immunofluorescence images of COL expressing FtsZ^WT^ (left) and M5 expressing FtsZ^G193D^ (right), obtained with an anti-FtsZ primary antibody. Scale bars: 1 µm. (b) Phase-contrast and fluorescence images showing the localization of sGFP-PBP2 in BCBPM073 expressing FtsZ^WT^ (left) and BCBRP003 expressing FtsZ^G193D^ (right). Scale bars: 1 µm. Download Figure S7, TIF file, 2.4 MB

Video S1  *S. aureus* M5 mutant cells exhibit a one-turn helical septum. The cell walls of the M5 mutant were labeled with the cell wall dye Van-FL and imaged by three-dimensional SIM. The image illustrates an example of a mutant M5 cell where the septum is placed as a one-turn helix. Scale bar: 1 µm. Download Video S1, AVI file, 0.6 MB

Table S1  (A) Strains and plasmids used in this study. (B) Primers used in this study. Table S1, DOCX file, 0.1 MB

Text S1  Growth conditions, strain construction, and techniques used for GTP hydrolysis assay, immunofluorescence assay, Western blotting, protein purification, and microscopy in this study. Download Text S1, DOCX file, 0.1 MB
