# Supplementary figures and images for: FtsZ-Dependent Elongation of a Coccoid Bacterium
Source: mBio. 2016 Sep 6;7(5):e00908-16. doi: 10.1128/mBio.00908-16 (PMC5013293; doi:10.1128/mBio.00908-16)

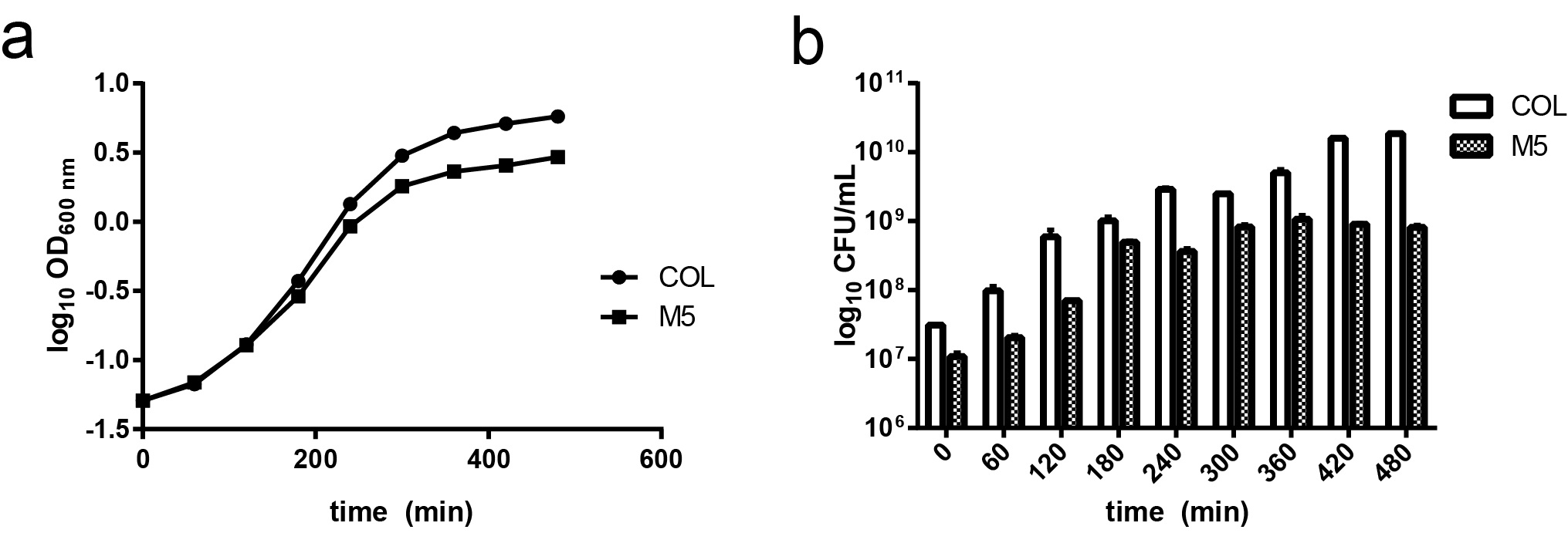

Supplement: Figure S1 — Analysis of COL wild-type and M5 mutant culture growth at 37°C. (a) Growth of COL and M5 cultures in TSB medium was monitored by recording the OD600 every hour. (b) The number of CFUs was also determined throughout the growth curve by plating appropriate dilutions of the growing COL and M5 cultures on solid tryptic soy agar. The graph shows the log10 number of CFU/ml versus time. Download [file mbo004162970sf1.tif]

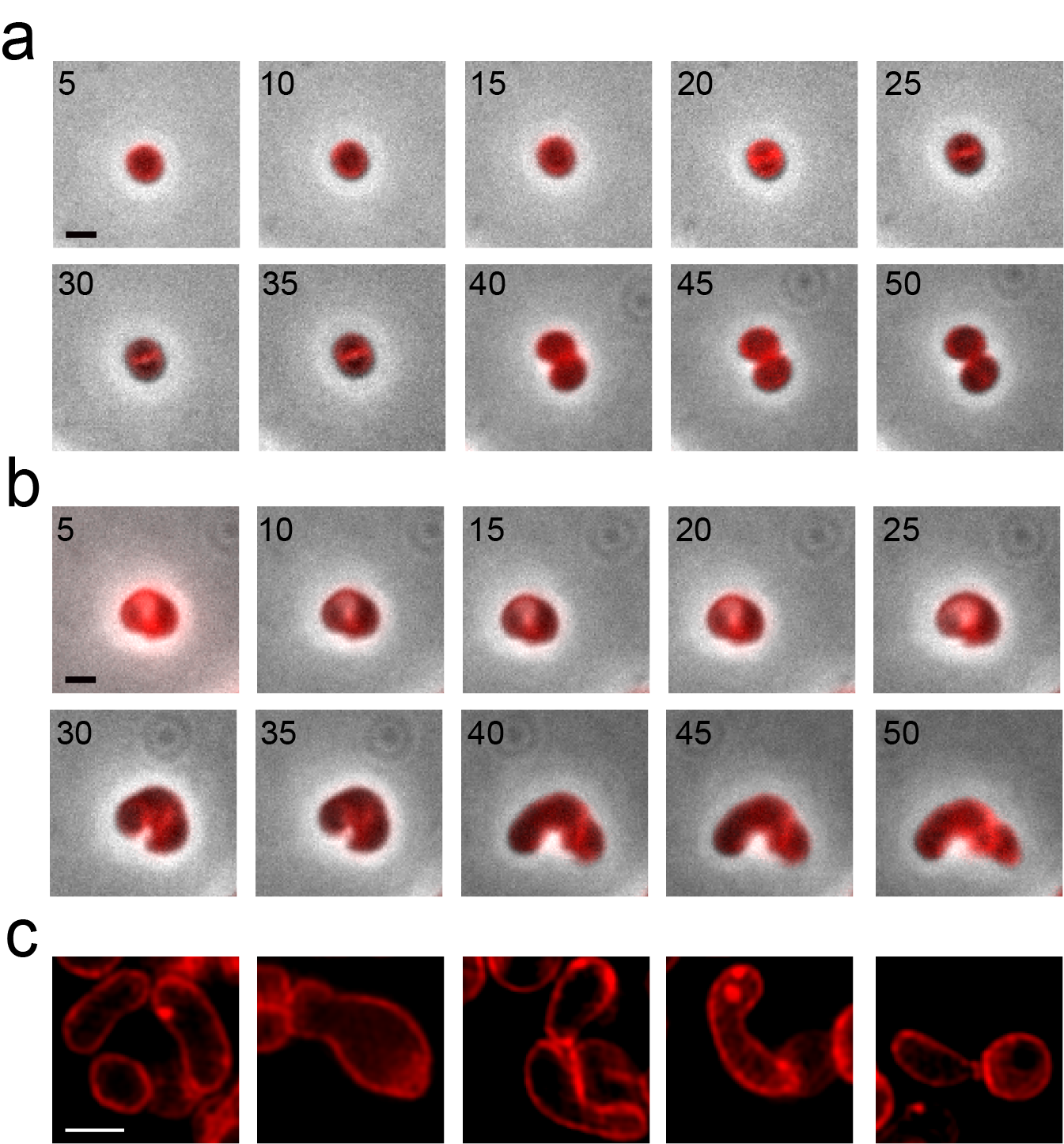

Supplement: Figure S2 — M5 mutant cell elongation observed by time-lapse microscopy and SIM. (a, b) Shown is time-lapse microscopy of COL wild-type (a) and M5 mutant (b) cells with examples of cells used to measure width/length ratios from round to elongated shapes plotted in Fig. 1b. Overlaid phase-contrast and Nile red-stained images are shown. Images were taken every 5 min. (c) Examples of elongated M5 cells without a septa stained with the membrane dye Nile red and observed by SIM. Scale bars: 1 µm. Download [file mbo004162970sf2.tif]

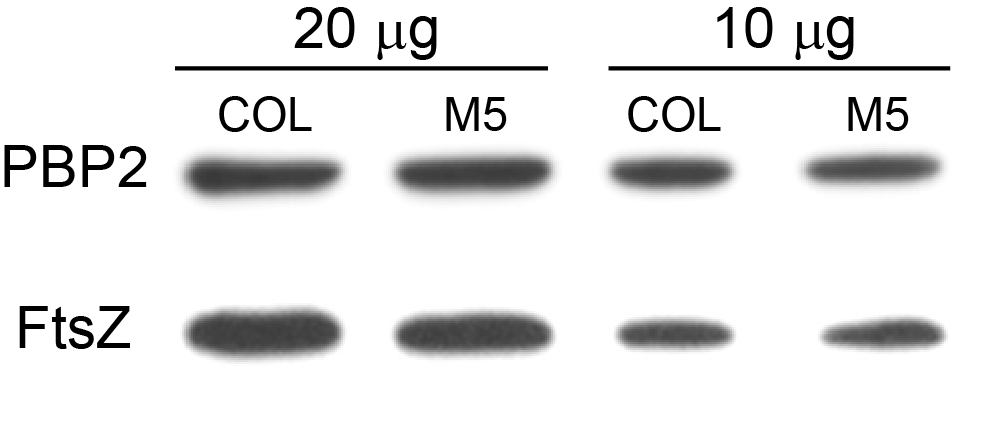

Supplement: Figure S3 — FtsZG193D levels in M5 cells are similar to FtsZWT levels in COL cells. Western blot analysis shows similar levels of FtsZ protein in COL and M5 cells. Twenty micrograms (first two lanes) or 10 µg (last two lanes) of total protein in crude cell extracts was loaded into the gel. PBP2 was used as an internal control. Download [file mbo004162970sf3.tif]

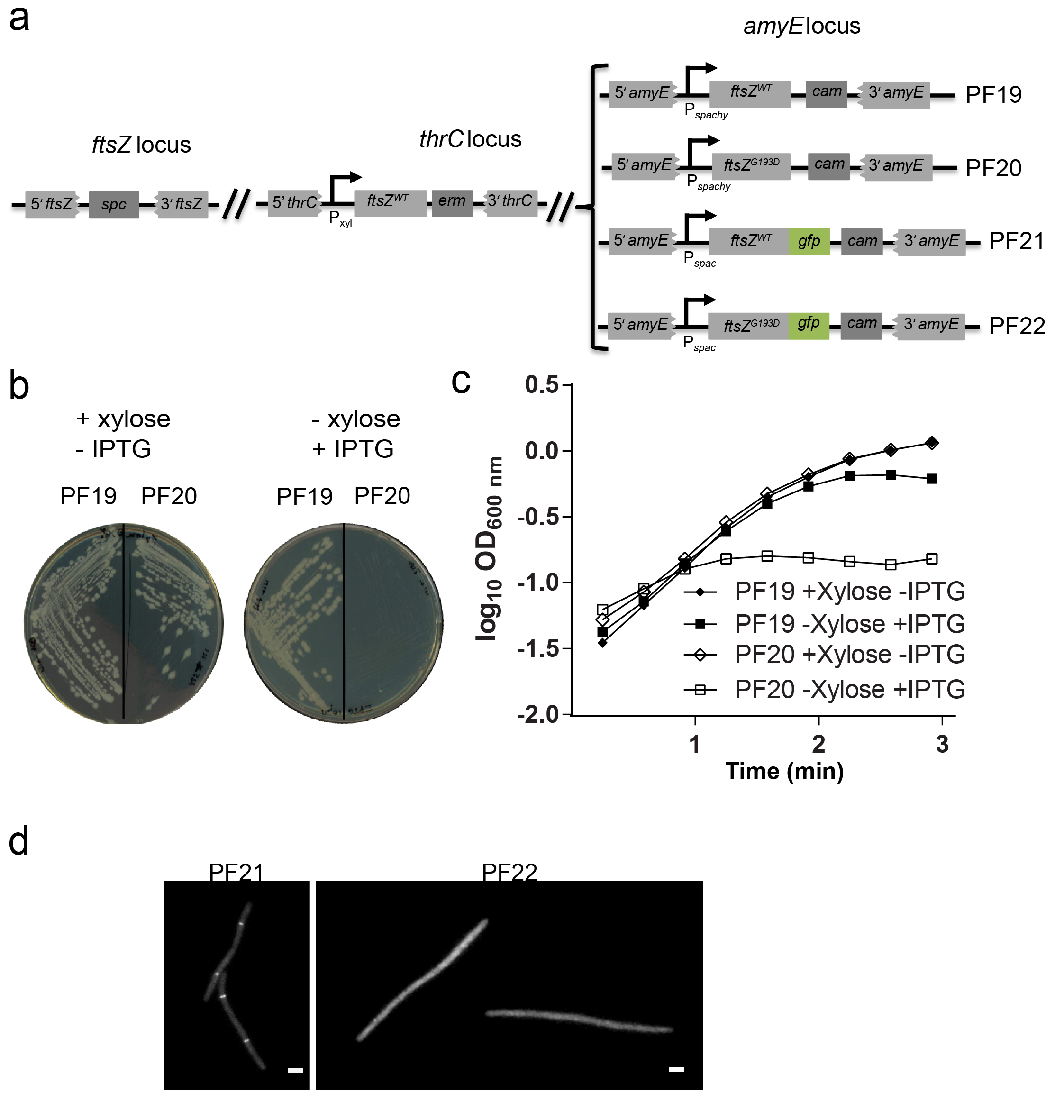

Supplement: Figure S4 — The FtsZG193D mutation renders FtsZ nonfunctional in B. subtilis. (a) Schematic representation of the genotypes of strains PF19, PF20, PF21, and PF22 used in this study. (b) Strains PF19 and PF20 were streaked onto plates supplemented with either 0.5% (wt/vol) xylose (left) or 10 µM isopropyl-β-d-thiogalactopyranoside (IPTG) (right) to induce the expression of the ftsZ alleles controlled by the respective promoters, as indicated in panel a. No differences in growth between strains PF20 and PF19 expressing only FtsZWT (in the presence only of xylose) were observed. However, strain PF20 was not viable when expressing FtsZG193D as the only source of FtsZ in the cell (in the presence only of IPTG). (c) Growth of PF20 and PF19 was measured in either LB plus xylose (0.2% [wt/vol], diamonds) or LB plus IPTG (100 µM, squares), confirming that cells expressing only FtsZG193D are not viable. (d) FtsZG193D-GFP localizes as a diffuse cytoplasmic signal in B. subtilis and cannot form Z rings. Cells of strains PF21 (left) and PF22 (right) were grown in LB plus IPTG (100 µM) to express FtsZWT or FtsZG193D, respectively, mounted on an agarose pad, and imaged by epifluorescence microscopy. Scale bars: 2 µm. Download [file mbo004162970sf4.tif]

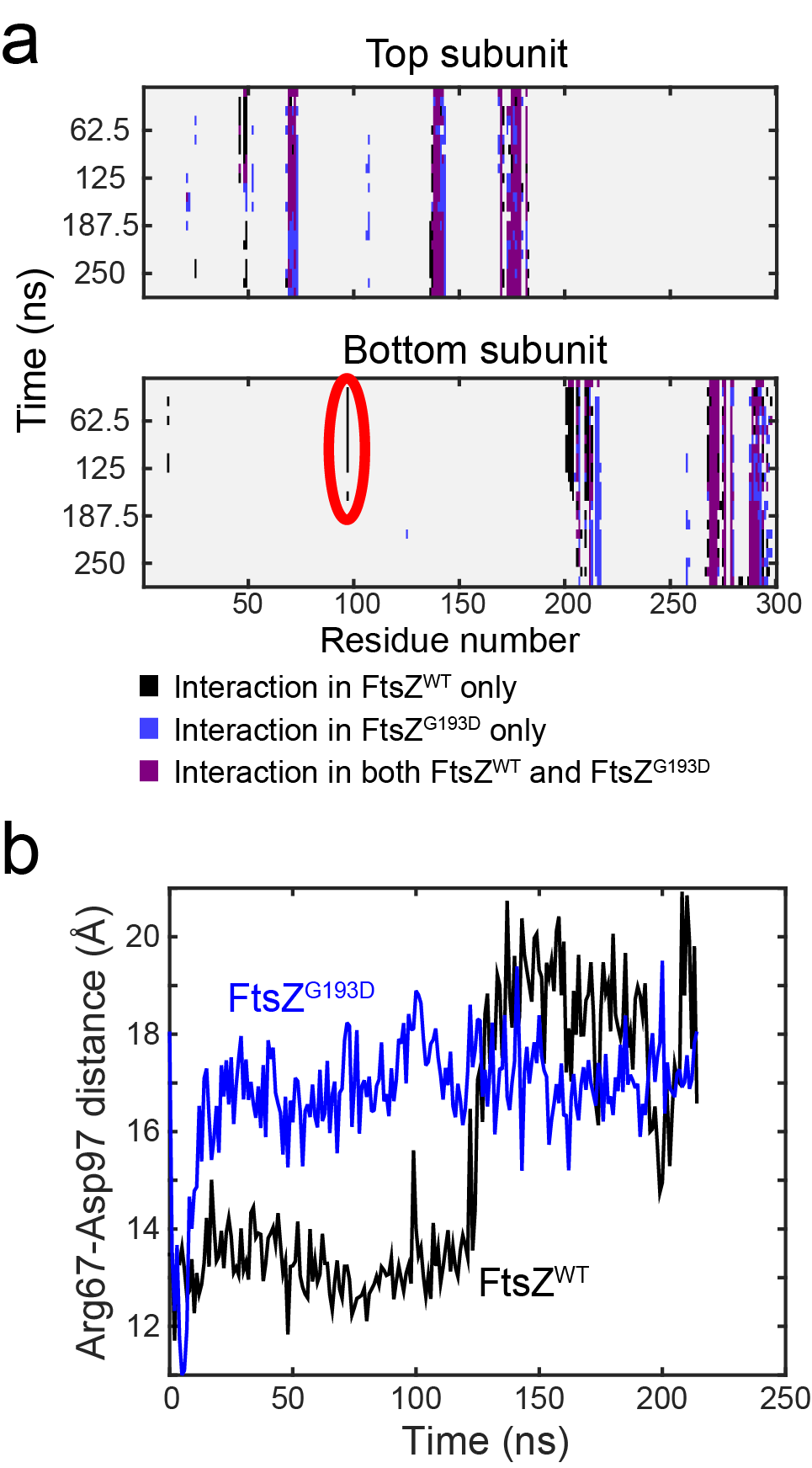

Supplement: Figure S5 — Interfacial interactions differ in FtsZWT and FtsZG193D and between the nontwisted and twisted states. (a) All of the residues that interact with the opposite subunit (defined as being within 5 Å of another residue) were identified in each frame of the simulations. Shown are the interactions in 12.5-ns blocks. Black, specific to the nontwisted state of the wild type, with an interaction in the first 100 ns and no interaction after 150 ns (and no interaction throughout the FtsZG193D simulation); purple, generally present in twisted states (always interacting in FtsZG193D and after 150 ns for FtsZWT); blue, specific to FtsZG193D. Red oval highlights Asp97. (b) Shown is the distance between the centers of mass of Arg67 and Asp97 spanning the dimer interface. The salt bridge was rapidly broken in the FtsZG193D dimer simulation. In the FtsZWT dimer simulation, the salt bridge briefly destabilized at t = ~100 ns and then broke for the remainder of the simulation at around t = 120 ns, mimicking the trajectory of polymer twist (Fig. 2b). Download [file mbo004162970sf5.tif]

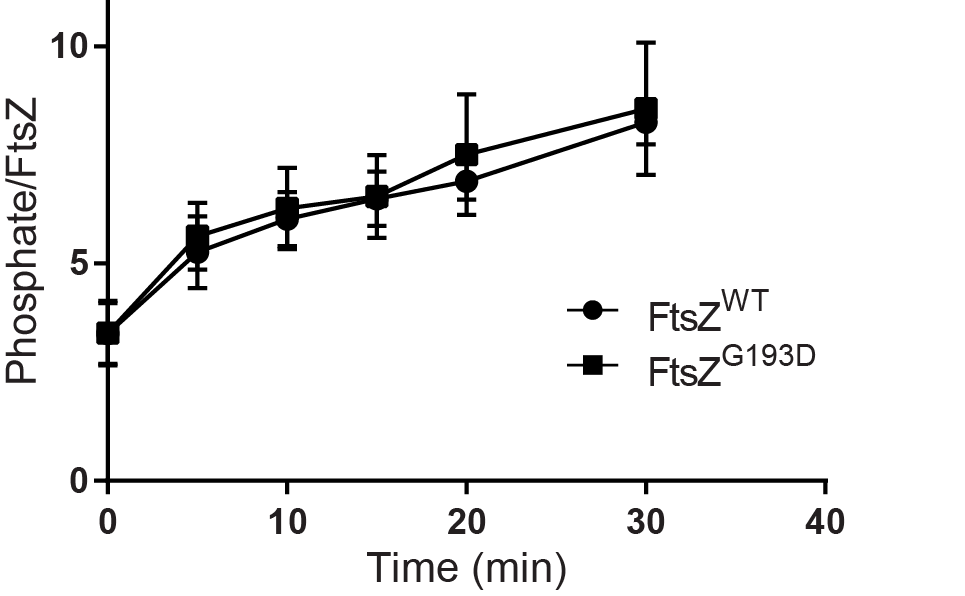

Supplement: Figure S6 — The FtsZG193D mutation does not affect GTP hydrolysis. Shown is the average number of phosphate molecules released per FtsZWT (circles) or FtsZG193D (squares) molecule. Average values are from four independent assays, and error bars represent standard deviations. Download [file mbo004162970sf6.tif]

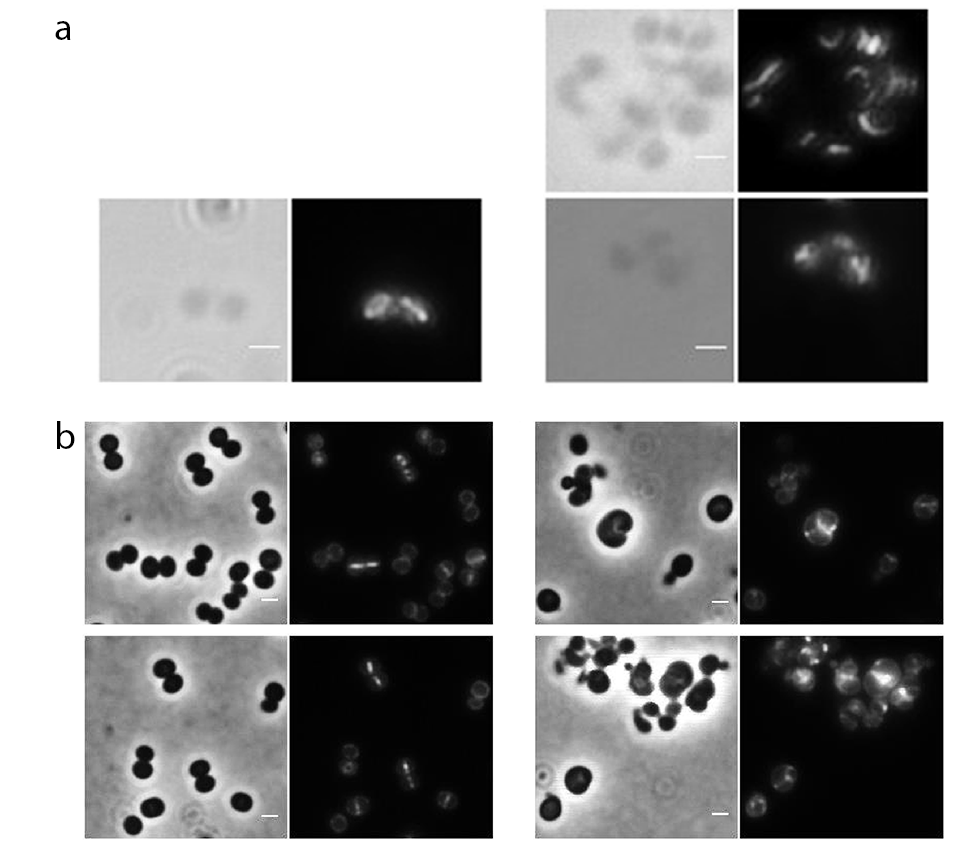

Supplement: Figure S7 — FtsZG193D and PBP2 do not form a mid-cell ring in S. aureus. (a) Phase-contrast and immunofluorescence images of COL expressing FtsZWT (left) and M5 expressing FtsZG193D (right), obtained with an anti-FtsZ primary antibody. Scale bars: 1 µm. (b) Phase-contrast and fluorescence images showing the localization of sGFP-PBP2 in BCBPM073 expressing FtsZWT (left) and BCBRP003 expressing FtsZG193D (right). Scale bars: 1 µm. Download [file mbo004162970sf7.tif]
