# Supplementary material for: FtsZ-Dependent Elongation of a Coccoid Bacterium
Source: mBio. 2016 Sep 6;7(5):e00908-16. doi: 10.1128/mBio.00908-16 (PMC5013293; doi:10.1128/mBio.00908-16)
Supplement: Table S1 — (A) Strains and plasmids used in this study. (B) Primers used in this study. [file mbo004162970st1.docx]

**Supplementary Table 1A:** Strains and plasmids used in this study

|  | **Relevant Characteristics** | **Source/**  **Reference** |
| --- | --- | --- |
| ***E. coli*** |  |  |
| DC10B | Δ*dcm* in the DH10B background; Dam methylation only | (1) |
| BL21(DE3) | *E. coli* B F^-^ *dcm* *ompT* *hsdS*(r_B_ ^-^ m_B_ ^-^) *gal* λ (DE3) | Stratagene |
| BL21(DE3)pCXZ_SA_ | BL21DE3 transformed with pCXZ_SA_; Amp^r^ | This study |
| BL21(DE3)pCXZ-*g578a* | BL21DE3 transformed with pCXZ-*g578a*; Amp^r^ | This study |
| ***S. aureus*** |  |  |
| RN4220 | MSSA strain. Mutagenized strain derived from NCTC8325-4 that accepts foreign DNA | R. Novick |
| COL | MRSA strain | (2) |
| M5 | COL with *ftsZg578a* mutation encoding FtsZG193D | (3) |
| BCBPM073 | COL*pbpB::sgfp-pbpB* | (3) |
| BCBRP003 | COL*ftsZg578a* *pbpB::sgfp-pbpB* | This study |
| BCBAJ020 | COLΔ*spa*::P_spac_-*ftsZ*-*5aa*-*cfp* *lacI* | (3) |
| BCBRP004 | COLΔ*spa*::P_spac_-*ftsZg578a*-*5aa*-*cfp* *lacI* | This study |
| BCBRP005 | COL*ftsZg578a*Δ*spa*::P_spac_-*ftsZg578a*-*5aa*-*cfp* *lacI* | This study |
| BCBAJ012 | COL*ezrA::ezrA-mCherry* | (4) |
| BCBRP006 | COL*ftsZg578a ezrA::ezrA-mCherry* | This study |
| ***B. subtilis*** |  |  |
| 168 | Wild-type strain | Lab collection |
| BW121 | *ftsZ::spc^r^, thrC::P_xyl_ -ftsZ erm^r^* | (5) |
| PL950 | *amyE::P_spachy_-ftsZ cat^r^* | (5) |
| PAL1114 | *amyE::P_spac_-ftsZ-gfp cat^r^* | (5) |
| BDA950 | 168 *amyE::P_spachy_-ftsZ cat^r^* | This study |
| PF17 | 168 *amyE::P_spachy_-ftsZ^G193D^ cat^r^* | This study |
| PF19 | BW121 *amyE::P_spachy_-ftsZ cat^r^* | This study |
| PF20 | BW121 *amyE::P_spachy_-ftsZ^G193D^ cat^r^* | This study |
| PF21 | BW121 *amyE::P_spac_-ftsZ-gfp cat^r^* | This study |
| PF22 | BW121 *amyE::P_spac_-ftsZ^G193D^-gfp cat^r^* | This study |
| **Plasmids** |  |  |
| pCXZ_BS_ | pJF118HE plasmid that contains P_tac_-*ftsZ* from *B. subtilis*; Amp^r^ | (6) |
| pCXZ_SA_ | pJF118HE plasmid that contains *bla* P_tac_-*ftsZ* from *S. aureus*; Amp^r^ | This study |
| pCXZ-*g578a* | pJF118HE plasmid that contains *bla* P_tac_-*ftsZg578a* from *S. aureus*; Amp^r^ | This study |
| pMAD | *E. coli* – *S. aureus* shuttle vector with the *bgaB* gene encoding a ß-galactosidase and thermosensitive origin of replication for gram-positive bacteria, Amp^r^ Erm^r^ | (7) |
| pMAD*ftsZg578a* | pMAD derivative used for replacement of *ftsZ* for *ftsZg578a,* Amp^r^  Erm^r^ | This study |
| pMUTINCFPKan | Integrative vector for C-terminal CFP fusions; Amp^r^, Kan^r^ | (8) |
| pBCBRP001 | pMUTINKan derivative containing *ftsZg578a*-*cfp*;  Amp^R^ Kan^r^ | This study |
| pBCB13 | pMAD derivative with P*_spac_* *lacI* between up- and downstream regions of the *spa* gene, Amp^r^  Erm^r^ | (9) |
| pBCB13-*ftsZg578a-cfp* | pBCB13 derivative containing P*_spac_*-*ftsZg578a-5aa-cfp lacI;* Amp^r^  Erm^r^ | This study |

**Supplementary Table 1B:** Primers used in this study

| **Primer Name** | **Sequence (5’-3’)** |
| --- | --- |
| ftsZP1 | tactcCCCGGGggccaataaaactaggagg |
| ftsZP2 | tgcacAGATCTATTAACCGATTAACGTCTTG |
| ftsZP3 | ATCGTGGGATCCTCTATTTGAATGATTATTG |
| ftsZP4 | ATCGTGGAATTCAATAACTTTGAAAAACTTAAATG |
| ftsZP5 | gctgcGGTACCggccaataaaactaggagg |
| ftsZP6 | GCTGCggtaccGGAGGCGCCGCAGGAacgtcttgttcttcttgaacg |
| ftsZP7 | tactcCCCGGGggccaataaaactaggagg |
| ftsZP8 | GCTGCCTCGAGTTACTTGTACAGCTCGTCCATGCCGAG |
| AC1257 | AAACCTCTTTACTGCCGTTATTCG |
| AC1258 | TCCCGTCTAGCCTTGCCCTCAATGG |
| PF37 | ACTTCGCCAAGATGTTCAAGGTATTTCTGACTTGA |
| PF38 | AGAAATACCTTGAACATCTTGGCGAAGTACGTTA |

Underlined sequences correspond to restriction sites.
