# Supplementary material for: FtsZ-Dependent Elongation of a Coccoid Bacterium
Source: mBio. 2016 Sep 6;7(5):e00908-16. doi: 10.1128/mBio.00908-16 (PMC5013293; doi:10.1128/mBio.00908-16)
Supplement: Text S1 — Growth conditions, strain construction, and techniques used for GTP hydrolysis assay, immunofluorescence assay, Western blotting, protein purification, and microscopy in this study. Download [file mbo004162970s1.docx]

**Supplementary MATERIALS AND METHODS**

**Bacterial strains and growth conditions**

*E. coli* and *B. subtilis* strains were grown on Luria Bertani agar (LB agar, Difco) or in Luria Bertani broth (LB broth, Difco). *S. aureus* strains were grown on tryptic soy agar (TSA, Difco) or in tryptic soy broth (TSB, Difco) at 37°C with aeration. The medium was supplemented, when required, with appropriate antibiotics (erythromycin 10 μg mL^-1^; kanamycin 50 μg mL^-1^; Sigma), with 5-bromo-4-chloro-3-indolyl β-D-galactopyranoside 100 μg mL^-1^ (X-Gal; Apollo Scientific) or with isopropyl-β-D-thiogalactopyranoside 0.5 mM (IPTG; Apollo Scientific). For *B. subtilis* strains, the medium was supplemented when required with antibiotics (spectinomycin 100 µg mL^-1^, chloramphenicol 10 µg mL^-1^ and erythromycin 5 µg m^-1^), 0.1 mM IPTG (VWR) and 0.2 % (w/v) xylose (Sigma).

**Super-resolution structured illumination microscopy (SIM)**

SIM imaging was performed using a Plan-Apochromat 63x/1.4NA oil DIC M27 objective, in an Elyra PS.1 microscope (Zeiss). Images were acquired using five phase shifts, either three or five grid rotations, with a 34-µm grating period for the 561-nm laser (100 mW), 28-µm period for the 488-nm laser (100 mW), and 23-µm period for the 405-nm laser (50 mW). Images were acquired using a sCMOS Pco.edge 5.5 camera and reconstructed using the ZEN software (black edition, 2012, version 8.1.0.484) based on a structured illumination algorithm (10) using synthetic and channel-specific optical transfer functions. ZEN software algorithms were used for channel alignment.

**Preparation of *S. aureus* cells for transmission electron microscopy (TEM)**

Embedding and thin sectioning of *S. aureus* cells was performed as described previously (4). Briefly, cells were harvested in exponential phase (OD_600_ _nm_ = 0.6), and washed with 0.1 M of sodium cacodylate buffer pH 7.4. Cell fixation was performed using 2.5% of glutaraldehyde in 0.1 M of sodium cacodylate buffer pH 7.4 followed by 1% osmium tetroxide. The fixed cells were rinsed to remove fixative and then dehydrated using increasing concentrations of acetone. After two final washes with 100% acetone, the cells were infiltrated with Spurr’s resin, transferred to 60 °C for polymerization, and the resulting blocks were thin-sectioned using an ultramicrotome (Reichert). Thin sections, ca. 80 nm thick, were picked up on un-coated 200 mesh copper grids and stained using uranyl acetate and lead nitrate (11). Thin sections were viewed in a Philips CM120 electron microscope and images were taken using an AMT ER50 5 megapixel CCD camera (Advanced Microscopy Techniques Corp., Danvers, MA) at magnifications ranging from 5,000 to 52,000x.

**Preparation of *S. aureus* cells for scanning electron microscopy (SEM)**

Cells were harvested in exponential phase, resuspended in fixative solution (2.5% glutaraldehyde in 0.2 M sodium cacodylate buffer, pH 7.4), deposited on glass discs (Marienfeld) and kept for one week at 4°C. The fixative solution was subsequently removed and the cells were washed three times with the sodium cacodylate solution. The sample was progressively dehydrated by immersion in a graded series of ethanol (50% - 100%) and then mounted on aluminium stubs with carbon adhesive discs (Agarscientific). The sample was critical-point dried under CO_2_ and sputter coated with gold-palladium (Polaron SC7640) for 200 s at 10 mA. SEM observations were performed using secondary electron images (2 kV) with a Hitachi S4500 instrument at the Microscopy and Imaging Platform (Micalis, B2HM, Massy, France) of the INRA research centre of Jouy-en-Josas (France).

**Construction of *Bacillus subtilis* *ftsZ* mutants**

To construct *B. subtilis* strains expressing the *ftsZg578a,g579t* allele, we used strain PL950 (12), which contains an additional copy of the wild-type *ftsZ* allele at the ectopic *amyE* locus under control of the IPTG-inducible promoter P*_spachy_*, and strain BW121 (12), which has its native *ftsZ* gene deleted and expresses FtsZ only from an ectopic *ftsZ* copy placed in the *thrC* locus under the control of a xylose-inducible promoter (P*_xyl_*). Similarly, we used strain PAL1114 (5) which contains a copy of a functional wild-type *ftsZ-gfp* fusion at the *amyE* locus, under control of the IPTG-inducible promoter P*_spac_*. We amplified the *amyE* locus from PL950 and PAL1114 chromosomal DNA, using primer pairs AC1257, PF38 and AC1258, PF37 (Table S1B), generating, in each case, two fragments with a 28-bp overlap that included the *g578a,g579t* mutation in either *ftsZ* or *ftsZ-gfp*. In each case, the overlapping fragments were ligated following a standard Gibson assembly protocol (13). Strains PF17 and PF20 were obtained by directly transforming wild-type strain 168 and strain BW121, respectively, with the resulting PCR products, and strains BDA950 and PF19 were obtained by transforming strains 168 and BW121, respectively, with a control PCR fragment that did not contain the mutation. Likewise, strains PF22 and PF21 were obtained by transforming strain BW121 with a PCR product containing either the *ftsZg578a,g579-gfp* or *ftsZ-gfp* allele, respectively. Transformations were performed using a standard *B. subtilis* transformation protocol for linear DNA fragments (14).

**Lethality analysis of the FtsZ^G193D^ mutation in *B. subtilis***

To assess the lethality of the FtsZ^G193D^ mutation, we examined the growth phenotypes of strains PF19 (*ftsZ::spc, thrC::P_xyl_-ftsZ erm, amyE::P_spachy_-ftsZ cat*) and PF20 (*ftsZ::spc, thrC::Pxyl-ftsZ erm, amyE::P_spachy_-ftsZ^G193D^ cat*). Strains PF19 and PF20 were streaked out on LB-agar plates supplemented with either 0.5% xylose (w/v) or 10 mM IPTG to induce expression of the *ftsZ* genes controlled by the respective inducible promoters. To determine growth rates, overnight PF19 and PF20 cultures in LB + 0.2% xylose were diluted to an OD_600 nm_ of 0.005 in the same medium, and grown to an OD_600 nm_ of 0.1. Culture samples were then washed and diluted ten-fold in either LB + xylose (0.2% w/v) or LB + IPTG (100 µM), and OD_600 nm_ was followed for 3 h.

**Localization of FtsZ^G193D^-GFP in *B. subtilis***

Cells from overnight liquid cultures in LB supplemented with xylose (0.2% w/v) of merodiploid strains PF21 and PF22 were diluted in fresh LB supplemented with xylose (0.2% w/v) until OD_600 nm_ = 0.1, then washed and diluted ten-fold in LB + 100 µM IPTG. 2h after this dilution, cells were mounted on an agarose pad and imaged by epifluorescence microscopy on an inverted microscope (Nikon Ti-E) using a 488-nm laser and an exposure time of 100 ms.

**Protein purification**

To produce *S. aureus* FtsZ^WT^ and FtsZ^G193D^ untagged proteins in *E. coli* BL21(DE3), DNA fragments containing the *ftsZ* and *ftsZg578a* alleles were amplified from the COL and M5 genomes, respectively, using primers ftsZP3 and ftsZP4. These PCR fragments were digested with BamHI and EcoRI, and cloned into the pCXZ_BS_ expression plasmid (6) previously digested with BamHI/EcoRI to remove the *B. subtilis* *ftsZ* allele. The resulting plasmids (pCXZ_SA_ and pCXZ-*g578a*) were sequenced and transformed into competent BL21(DE3) cells to generate strains BL21(DE3)pCXZ_SA_ and BL21(DE3)pCXZ-*g578a*, respectively.

*S. aureus* FtsZ^WT^ protein was purified from strain BL21(DE3)pCXZ_SA_ as previously described, with minor modifications (17, 18). Briefly, FtsZ was precipitated from a membrane-free cell extract with a 50% ammonium sulphate cut, resuspended in a solution of 50 mM Tris/HCl, 1 mM EDTA, and 10% (v/v) glycerol (pH 8.5), and loaded onto a Source 30Q anion exchange column (GE Life Sciences). Elution of the protein was performed with a 50-500 mM KCl gradient. Purified FtsZ was dialysed against storage buffer (20 mM Tris/HCl, 1 mM EGTA, 2.5 mM magnesium acetate, 10% (v/v) glycerol, and 50 mM KCl, pH 7.9), frozen in liquid nitrogen, and stored at -80 °C.

*S. aureus* FtsZ^G193D^ protein was purified from strain BL21(DE3)pCXZ-*g578a* as described above with the addition of an extra gel-filtration step after the anion-exchange chromatography using a Superdex 200 HiLoad 16/60 column equilibrated and eluted with storage buffer. Purified FtsZ^G193D^ aliquots were frozen in liquid nitrogen and stored at -80 °C. FtsZ protein concentrations were determined using the BCA protein assay kit (Pierce, Life Technologies).

**Transmission electron microscopy (TEM) of FtsZ polymers**

TEM of FtsZ^WT^ and FtsZ^G193D^ polymers was performed as previously described (19). Briefly, 5 µM FtsZ^WT^ or FtsZ^G193D^ protein were individually incubated in polymerization buffer (50 mM HEPES-NaOH pH 7.5, 300 mM KCl, 10 mM MgCl_2_) for 5 min at 30 ºC. Polymerization was initiated by adding GTP to a final concentration of 2 mM, after which the samples were incubated at 30 ºC for 10 min. Per reaction, two microliters were placed on individual glow-discharged 400-mesh carbon-coated copper grids and immediately blotted dry with filter paper. Samples were negatively stained with 2 µL of 2% uranyl acetate. Grids were viewed using a Philips CM120 electron microscope equipped with a LaB_6_ filament operating at 120 kV. Images were recorded with a Gatan 4000 SP 4K slow-scan CCD camera. The lengths of more than 70 polymers were measured using ImageJ. Statistical analyses of length differences between FtsZ^WT^ and FtsZ^G193D^ polymers were analysed with GraphPad Prism 5 using the Mann-Whitney test for non-normal distributions.

**GTP hydrolysis assays**

GTP hydrolysis was measured as previously described (19), using the malachite green phosphate assay kit (Bioassays) to measure phosphate release over time. Either FtsZ^WT^ or FtsZ^G193D^ proteins (10 µM) were incubated in polymerization buffer supplemented with 2 mM GTP at 30 ºC (final reaction volume of 40 µL). At different time points, 20 µL of each reaction were mixed with 20 µL of malachite green working reagent (Bioassays) to stop the reaction. After incubation for 30 min at room temperature, the OD_630 nm_ was recorded using a PowerWave HT microplate spectrophotometer (BioTek). Released phosphate was calculated using an internal phosphate standard. Phosphate release over time, from four experiments, was plotted using GraphPad Prism 5.

**Construction of *S. aureus* mutants**

To replace wild-type *ftsZ* with an *ftsZ* gene containing the *g578a* mutation encoding FtsZ^G193D^ in the background of *S. aureus* strains encoding fluorescent fusions of sGFP-PBP2 (strain BCBPM073 (3)) or EzrA-mCherry (strain BCBAJ012 (4)), we first amplified an 1172-kb DNA fragment, using the M5 genome DNA as a template and primers ftsZP1 and ftsZP2. This DNA fragment contains a G to A point mutation in *ftsZ*, at nucleotide position 578 (*ftsZg578a*). This DNA fragment was then cloned into the pMAD plasmid (7), giving rise to pMAD*ftsZg578a* which was confirmed by sequencing. This plasmid was electroporated into RN4220 (15) and subsequently transduced into strains BCBPM073 and BCBAJ012 using phage 80α (16). Integration and excision of pMAD*ftsZg578a* from the genome was performed as previously described (7) and colonies in which native *ftsZ* was replaced by the *ftsZg578a* allele were selected by replica plating on TSA supplemented or not with 25 μg mL^-1^ of oxacillin as the M5 mutant is susceptible to this antibiotic (3). Strains that did not grow in the presence of oxacillin were sequenced to confirm *ftsZ* replacement by the *ftsZg578a* allele. Strains derived from BCBPM073 and BCBAJ012 that contained the *ftsZg578a* allele were named BCBRP003 and BCBRP006, respectively.

For the construction of a strain expressing an FtsZ^G193D^-CFP fusion from the ectopic *spa* locus, a PCR fragment encompassing the *ftsZg578a* mutation was amplified from M5 genomic DNA using primers ftsZP5 and ftsZP6 (which encodes a 5 amino acid linker), cloned into pMUTINCFPKan (8) and sequenced, generating the pBCBRP001 plasmid. The full *ftsZg578a-5aa-cfp* construct was then amplified from the pBCBRP001 plasmid using primers ftsZP7 and ftsZP8 and cloned into the pBCB13 plasmid (9), downstream of the IPTG-inducible P*_spac_* promoter. The resulting plasmid (pBCB13-*ftsZg578a-cfp*) was confirmed by sequencing. This plasmid was then electroporated into RN4220 (15), grown at 30 ºC with erythromycin selection, and subsequently transduced into COL strain using phage 80α (16). The replacement of the *spa* gene with the P*_spac_*-*ftsZg578a-cfp* construct was performed as previously described (9) and confirmed by PCR. The resulting COL strain expressing *ftsZg578a*-*cfp* from the *spa* locus was named BCBRP004. Since this strain still has a wild-type *ftsZ* copy at the native locus, we transduced the pMAD*ftsZg578a* construct into BCBRP004 and substituted the native *ftsZ* gene by the *ftsZg578a* allele, as described above, giving rise to strain BCBRP005.

**Epifluorescence Microscopy**

BCBPM073, BCBRP003, BCBAJ020 and BCBRP005 strains were grown overnight in TSB and diluted 1/500 in either TSB (BCBPM073 and BCBRP003 strains) or TSB supplemented with 0.1 mM of IPTG (BCBAJ020 and BCBRP005 strains) and incubated at 37 ºC. At mid-exponential phase (OD_600 nm_ of 0.6), cells (1 mL of each culture) were pelleted by centrifugation, re-suspended in 20 µL of phosphate-buffered saline (PBS) and 1 µL was placed on microscopy slide covered with a thin layer of 1.2% agarose in PBS. Images were obtained using a Zeiss Axio Observer.Z1 microscope equipped with a photometrics CoolSNAP HQ2 camera (Roper Scientific) using Metamorph software (Molecular Devices).

**Immunofluorescence microscopy**

Immunofluorescence labelling of FtsZ was performed as previously described (20). Briefly, *S. aureus* COL and M5 strains were grown to an OD_600 nm_ of 0.6, culture samples (10 mL) were centrifuged and the pellet was fixed with 1 mL of Histochoice (Amresco). Cells were washed three times with PBS and re-suspended in 500 μL of GTE buffer (50 mM glucose, 20 mM Tris-HCl, pH 7.5, 10 mM EDTA). A gentle lysis was performed using lysostaphin (Sigma) at a final concentration of 10 µg mL^-1^ for 1 minute. During the incubation with lysostaphin, 25 µL of each culture were placed on top of polylysine-treated slide wells. After the incubation period with lysostaphin, cells were washed three times with GTE, air dried, rehydrated with PBS, and blocked with 2% bovine serum albumin (BSA, Sigma) in PBS for 15 min. Cells were then incubated overnight at 4 °C with anti-FtsZ primary antibody, which was added in consecutive two-fold dilutions from 1:800 to 1:3,600. The following day, cells were washed eight times with PBS and incubated with secondary antibody (Alexa Fluor 488 donkey anti-sheep IgG, Invitrogen diluted 1:500 in 2% BSA/PBS) in the dark for 1 h. Cells were again washed eight times with PBS and 1.5 µL of Vectashield mounting medium (Vector Laboratories) was added. Cells were visualized by epifluorescence microscopy.

**Western blot analysis**

Expression levels of FtsZ were analysed by western blotting, using a polyclonal anti-FtsZ specific antibody produced against *B. subtilis* FtsZ. A polyclonal anti-PBP2 antibody was used as an internal control (21). Samples were taken from cultures of COL and M5 grown until an OD_600 nm_ of 0.8. Cells were broken with glass beads in a Fast Prep FP120 (Thermo Electro Corporation) and debris was removed by centrifugation. The total protein content of the extracts was quantified by the Bradford method, using bovine serum albumin as a standard (BCA protein assay kit, Pierce). 10 µg and 20 µg of total protein from each sample were loaded onto an 10% SDS-PAGE gel and separated at 120 V. Proteins were then transferred to a Hybond-P Polyvinylidene fluoride (PVDF) membrane (GE Healthcare) using a semidry transfer cell (Bio-Rad). The membranes were cut to separate the region containing PBP2 and FtsZ. Each half of the membrane was blocked with blocking buffer (PBS; 5% milk; 0.5% Tween 20) for 1 h and incubated with either a polyclonal anti-PBP2 antibody (21) or with an anti-FtsZ antibody for 16 h at 4 °C. Membranes were washed three times with PBS-T (PBS containing 0.5% Tween 20) and incubated with secondary antibodies (HRP anti-rabbit diluted 1/100,000 for PBP2 detection, VWR; HRP anti-sheep diluted 1/50,000 for FtsZ detection, Pierce). The detection was performed using an ECL Plus Western blotting detection system (Amersham) according to the manufacturer’s guidelines.

**3D reconstructions using super-resolution structured illumination microscopy**

*S. aureus* overnight M5 cultures were diluted 1:200 in fresh media and incubated at 37ºC. One millilitre of each culture in exponential phase (OD_600 nm_ = 0.6) was incubated with the membrane dye Nile Red (10 μg mL^-1^, Invitrogen) and with the cell-wall dye Van-FL (1 μg mL^-1^, Invitrogen) mixed in a 1:1 (v:v) proportion with non-fluorescent vancomycin (1 μg mL^-1^, Sigma). Cells were harvested by centrifugation, resuspended in 20 μL of PBS and placed on top of a thin layer of 1.2% agarose in PBS mounted on a microscope slide. 3D reconstruction of SIM images was performed with a Zeiss Elyra PS.1 microscope, by acquiring z-stacks with 0.15 µm increments. 3D-SIM reconstructions were performed using the Zen software and a point spread function (PSF) based on theoretical models.

**Supplementary References**

1. **Monk IR, Shah IM, Xu M, Tan MW, Foster TJ.** 2012. Transforming the untransformable: application of direct transformation to manipulate genetically *Staphylococcus aureus* and *Staphylococcus epidermidis*. MBio **3**.

2. **Gill SR, Fouts DE, Archer GL, Mongodin EF, Deboy RT, Ravel J, Paulsen IT, Kolonay JF, Brinkac L, Beanan M, Dodson RJ, Daugherty SC, Madupu R, Angiuoli SV, Durkin AS, Haft DH, Vamathevan J, Khouri H, Utterback T, Lee C, Dimitrov G, Jiang L, Qin H, Weidman J, Tran K, Kang K, Hance IR, Nelson KE, Fraser CM.** 2005. Insights on evolution of virulence and resistance from the complete genome analysis of an early methicillin-resistant *Staphylococcus aureus* strain and a biofilm-producing methicillin-resistant *Staphylococcus epidermidis* strain. J Bacteriol **187:**2426-2438.

3. **Tan CM, Therien AG, Lu J, Lee SH, Caron A, Gill CJ, Lebeau-Jacob C, Benton-Perdomo L, Monteiro JM, Pereira PM, Elsen NL, Wu J, Deschamps K, Petcu M, Wong S, Daigneault E, Kramer S, Liang L, Maxwell E, Claveau D, Vaillancourt J, Skorey K, Tam J, Wang H, Meredith TC, Sillaots S, Wang-Jarantow L, Ramtohul Y, Langlois E, Landry F, Reid JC, Parthasarathy G, Sharma S, Baryshnikova A, Lumb KJ, Pinho MG, Soisson SM, Roemer T.** 2012. Restoring methicillin-resistant *Staphylococcus aureus* susceptibility to beta-lactam antibiotics. Sci Transl Med **4:**126-135.

4. **Jorge AM, Hoiczyk E, Gomes JP, Pinho MG.** 2011. EzrA contributes to the regulation of cell size in *Staphylococcus aureus*. PLoS One **6:**e27542.

5. **Weart RB, Levin PA.** 2003. Growth rate-dependent regulation of medial FtsZ ring formation. J Bacteriol **185:**2826-2834.

6. **Wang X, Lutkenhaus J.** 1993. The FtsZ protein of *Bacillus subtilis* is localized at the division site and has GTPase activity that is dependent upon FtsZ concentration. Mol Microbiol **9:**435-442.

7. **Arnaud M, Chastanet A, Debarbouille M.** 2004. New vector for efficient allelic replacement in naturally nontransformable, low-GC-content, gram-positive bacteria. Appl Environ Microbiol **70:**6887-6891.

8. **Veiga H, Jorge AM, Pinho MG.** 2011. Absence of nucleoid occlusion effector Noc impairs formation of orthogonal FtsZ rings during *Staphylococcus aureus* cell division. Mol Microbiol **80:**1366-1380.

9. **Pereira PM, Veiga H, Jorge AM, Pinho MG.** 2010. Fluorescent reporters for studies of cellular localization of proteins in *Staphylococcus aureus*. Appl Environ Microbiol **76:**4346-4353.

10. **Heintzmann R, Cremer CG.** 1999. Laterally modulated excitation microscopy: improvement of resolution by using a diffraction grating. Proceedings of the SPIE3568 (Optical Biopsies and Microscopic Techniques III)**:**185–196

11. **Reynolds ES.** 1963. The use of lead citrate at high pH as an electron-opaque stain in electron microscopy. J Cell Biol **17:**208-212.

12. **Weart RB, Lee AH, Chien AC, Haeusser DP, Hill NS, Levin PA.** 2007. A metabolic sensor governing cell size in bacteria. Cell **130:**335-347.

13. **Gibson DG, Young L, Chuang RY, Venter JC, Hutchison CA, Smith HO.** 2009. Enzymatic assembly of DNA molecules up to several hundred kilobases. Nat Methods **6:**343-345.

14. **Albano M, Hahn J, Dubnau D.** 1987. Expression of competence genes in *Bacillus subtilis*. J Bacteriol **169:**3110-3117.

15. **Veiga H, Pinho MG.** 2009. Inactivation of the SauI type I restriction-modification system is not sufficient to generate *Staphylococcus aureus* strains capable of efficiently accepting foreign DNA. Appl Environ Microbiol **75:**3034-3038.

16. **Oshida T, Tomasz A.** 1992. Isolation and characterization of a Tn551-autolysis mutant of *Staphylococcus aureus*. J Bacteriol **174:**4952-4959.

17. **Mukherjee A, Lutkenhaus J.** 1998. Dynamic assembly of FtsZ regulated by GTP hydrolysis. EMBO j **17:**462-469.

18. **Scheffers DJ.** 2008. The effect of MinC on FtsZ polymerization is pH dependent and can be counteracted by ZapA. FEBS Lett **582:**2601-2608.

19. **Krol E, Scheffers DJ.** 2013. FtsZ polymerization assays: simple protocols and considerations. J Vis Exp **81:**e50844.

20. **Pinho MG, Errington J.** 2003. Dispersed mode of *Staphylococcus aureus* cell wall synthesis in the absence of the division machinery. Mol Microbiol **50:**871-881.

21. **Reed P, Veiga H, Jorge AM, Terrak M, Pinho MG.** 2011. Monofunctional transglycosylases are not essential for *Staphylococcus aureus* cell wall synthesis. J Bacteriol **193:**2549-2556.
